# Epithelial Cell-Associated Galectin-3 Activates Human Dendritic Cell Subtypes for Pro-Inflammatory Cytokines

**DOI:** 10.3389/fimmu.2020.524826

**Published:** 2020-10-14

**Authors:** John T. Schroeder, Abiodun A. Adeosun, Anja P. Bieneman

**Affiliations:** The Department of Medicine, Division of Allergy and Clinical Immunology, Johns Hopkins Asthma and Allergy Center, Johns Hopkins University, Baltimore, MD, United States

**Keywords:** inflammation, lectin, cytokine, epithelial cell, fibrosis, cancer, cardiovascular disease

## Abstract

There is mounting evidence that galectin-3 is a prognostic and diagnostic biomarker associated with diverse diseases and conditions, including cancer, cardiovascular disease, autoimmunity, wound healing, allergic disease, and chronic inflammation in general. Yet, whether and exactly how galectin-3 may participate in the pathogenesis of these diseases remains poorly understood. Recently, we have linked the expression of galectin-3 on the A549 epithelial cell line –an adenocarcinoma, to the activation of human basophils for the release of histamine and secretion of IL-4 and IL-13. These responses proved dependent on cell-to-cell contact, basophil expression of IgE, were inhibited by n-acetyllactosamine, and were ablated when basophils were co-cultured with A549 clones lacking galectin-3 expression. While recombinant galectin-3 failed to activate basophils when in solution, microspheres expressing this lectin did so by mimicking the responses seen when using A549 cells. Given the IgE dependency of the basophil responses, and the fact that galectin-3 is long known to bind this immunoglobulin, we hypothesize that a similar mode of activation extends to other IgE-bearing cells. To investigate this possibility, we tested epithelial cell-associated galectin-3 for its capacity to activate human dendritic cells, including the plasmacytoid and myeloid subtypes as well as monocytes, all of which bind IgE. Indeed, results indicate that epithelial cell-associated galectin-3 activated these cells for robust production of TNF-α and IL-6 and up-regulated the expression of activation markers found on dendritic cells. Moreover, many of the same parameters previously observed for basophils applied to the findings herein, including evidence that matrix-bound galectin-3 (whether on epithelial cells or microspheres) facilitates this mode of activation. In contrast, IgE expression was dispensable for these galectin-3-dependent cytokine responses, implying that this lectin activates dendritic cells (and monocytes) by binding to a glycoprotein other than this immunoglobulin. Overall, these findings further demonstrate how galectin-3 mediates immune cell activation, providing novel insight into how this lectin may promote chronic inflammation underlying the pathogenesis of many diseases.

## Introduction

Crosslinking of the IgE receptor (FcεRI) on human basophils, and its αγ_2_ variant on dendritic cells (DC) and monocytes, is known to activate these cells to secrete pro-inflammatory cytokines. In particular, basophils secrete IL-4 and IL-13 that are central to the allergic diathesis (1). Plasmacytoid DC (pDC), myeloid DC (mDC), and monocytes are all reported to secrete TNF-α, IL-6, and IL-10 upon crosslinking IgE/FcεRI (2–6). Equally documented for DC are the impaired innate immune responses that ensue as a result of this mode of activation, which has led to recent clinical evidence that *in vivo* neutralization of IgE using omalizumab may very well restore this impairment (7, 8). Naturally, the interaction of allergen with specific IgE bound to FcεRI is the mode of stimulation most often associated with facilitating these responses. However, we recently reported evidence for a unique mode of IgE-dependent activation in basophils whereby these granulocytes released histamine and secreted large quantities of IL-4/IL-13 when co-cultured with the A549 epithelial cell (EC) line –an adenocarcinoma of lung origin (9). These responses proved dependent on cell-to-cell contact and on the expression of IgE by basophils. They were also inhibited by compounds targeting FcεRI-dependent signaling and by n-acetyllactosamine. By suppressing galectin-3 (Gal-3) expression in A549 cells using shRNA, and by using microspheres (MS) coupled with rhGal-3 protein (Gal-3-MS), we have more recently provided direct evidence that this lectin possesses the capacity to activate basophils (10). These findings in basophils have now lead us to hypothesize that Gal-3, including that associated with matrix cells (e.g., EC) activates DC subtypes through a similar Gal-3/IgE-dependent mechanism.

Importantly, of the 15 known mammalian galectins, Gal-3 and Gal-9 are the only two reported to bind IgE. In particular, Gal-3 was originally identified as an IgE-binding lectin and is unique among galectins by possessing a chimera structure that allows formation of oligomers (e.g., pentamers) capable of multivalent binding *via* carbohydrate recognition domains (CRDs) (11, 12). Indeed, early studies showed that Gal-3 activates RBL mast cells for serotonin release, presumably by facilitating IgE/FcεRI crosslinking (13). In contrast, Gal-9 has a tandem repeat structure with just two CRD domains, theoretically making it less likely to induce IgE/FcεRI crosslinking, especially if one of the CRDs is already associated with a glycoprotein. In fact, Gal-9 is reported to inhibit IgE-dependent activation, both *in vitro* and *in vivo* (14).

With regard to Gal-3 effects on DC, a number of studies have been conducted, but mostly using mouse knockouts deficient of this lectin. For example, Brand, et al. reported a number of immune cell alterations in the spleens of Gal-3 deficient mice during chronic infection, including decreased numbers of macrophages (15). More recently, Kouo, et al., while investigating the suppressive effects of Gal-3 on the anti-tumor responses of CD8^+^ T-cells, noted that Gal-3^-/-^ mice have increased numbers of circulating pDC, thus implying that Gal-3 limits the expansion of pDC (16). Similarly, other studies have investigated the impact of suppressing intracellular Gal-3 specifically in DC and/or macrophages and the consequences of doing so on the maturation and function of these immune cells. In particular, Chen, et al. recently used siRNA technology to knock down Gal-3 in human monocyte-derived DC, showing that this negatively impacted the capacity of these cells to respond to innate immune stimuli for pro-inflammatory cytokine production and to induce T cell responses (17).

In contrast to the above approaches, which have essentially investigated the impact of depleting intracellular Gal-3 in DC and/or monocytes, we have instead explored whether EC-associated Gal-3 activates these immune cells. Again, the rationale for conducting these experiments stemmed, in part, from our novel finding that cancer cell (A549)-associated Gal-3 activated human basophils. Thus, there is good impetus in determining whether other immune cells are similarly activated in this manner. For example, increased Gal-3 expression in lesion sites and/or elevated levels in plasma/serum have since been linked to many diseases, ranging from cancer to asthma (18). Identifying EC-associated Gal-3 as a novel stimulus of DC/monocytes, and possibly in an IgE-dependent manner recently shown for basophils, adds further insight into how this lectin might mediate the activation of various immune cells in a vast range of inflammatory conditions. Accordingly, we show evidence herein that DC are indeed stimulated by extracellular Gal-3 to produce pro-inflammatory cytokines (e.g., TNF-α and IL-6). Interestingly, this mode of activation is best facilitated by immobilized Gal-3, particularly that found associated with EC –a finding that is mimicked by coupling this lectin onto MS. And, while this Gal-3-dependent activation shares several parameters consistent with those previously identified with FcεRI/IgE crosslinking, results clearly reveal that IgE expression by DC is not a requirement. Nevertheless, these data are the first to identify EC-associated Gal-3 as a potent activator of human DC, inducing these cells to secrete large quantities of cytokines hallmark to the chronic inflammation underlying the pathogenesis of numerous diseases.

## Materials and Methods

### Special Reagents, Buffers, and Media

The following reagents were purchased: crystallized human serum albumin (Calbiochem-Behring Corp, La Jolla, CA); PIPES, FCS, and crystallized BSA (Sigma-Aldrich, Allentown, PA); MOPS (3-(*N*-morpholino)propanesulfonic acid) buffer (Honeywell-Fluka, Pittsburg, PA), gentamicin, IMDM, StemPro-34 serum free medium, and nonessential amino acids (Life Technologies, Inc, Grand Island, NY); Percoll (Pharmacia Biotec, Inc, Piscataway, NJ); rhIL-3 (Thermofisher, Grand Island, NY); rhGal-3 and rhGal-9 were purchased from Abcam Inc. (Cambridge, MA) and R&D Systems (Minneapolis, MN), respectively and reported to be ≥95% pure. Biological activity was additionally reported by the venders based on the ability of rhGal-3 to agglutinate human red blood cells at 2.5 μg/ml and for immobilized rhGal-9 to support the adhesion of Jurkat T cells at 0.75–4.5 μg/ml. Monoclonal anti-Gal-3 (clone B2C10) and IgG_1_ isotype (clone MOPC-21) control (Santa Cruz, Dallas, TX); Gal-3 ELISAs (e-Bioscience, San Diego, CA). Goat anti-IgG_1_-Alex647 and -Alex594 (both from Invitrogen, Eugene, OR); HLA-DR-PE, CD80-PE, CD86-PE, and CD40-PE (Becton Dickinson, San Jose, CA); rhFlt-3 ligand, rhTPO, rhSCF, and StemRegenin 1 (SR-1) (PeproTech, Rocky Hill, NJ); C-IMDM consisted of IMDM supplemented with 5% FCS, non-essential amino acids, L-glutamine, 10 μg/ml gentamicin, pH 7.4. The goat polyclonal anti-human IgE and the myeloma IgE (JK) were provided by Dr. Robert Hamilton, JHU. The SE44 IgE, which recognized a synthetic 15 amino acid peptide (R15K) has been described elsewhere (19) and was provided by Dr. Donald MacGlashan, Jr., JHU.

### A549 Cell Culture and Gal-3 Knockdown Using shRNA

A549 EC (American Type Culture Collection ATCC, Manassas, VA) were maintained in medium consisting of F-12K nutrient mixture –Kaigh’s Modification, with 10% heat-inactivated FBS and 1% penicillin streptomycin. Cultures were split twice weekly during the duration of these experiments, with no detectable changes in their capacity to activate DC. Seeding of EC for co-culture with basophils has been previously described (9) and was similarly done herein for DC co-cultures. Briefly, EC were plated into wells of 96-well plates in 0.100 ml of the medium. These were incubated up to 48h in a humidified incubator (37°C, 5% CO_2_) to allow cells to adhere and achieve ~70%–80% confluency prior to co-culture with pDC and mDC.

The generation of stable A549 clones deficient of Gal-3 protein is recently described (10). In brief, shRNA technology using a Mission^®^ Lentiviral Transduction kit and protocol (Sigma-Aldrich, St. Louis, MO) was used to produce clones (1E2 and 1B10) showing >95% reduction in total Gal-3 protein, including surface Gal-3. This approach also produced a control clone (3G8) whereby ~50% of total Gal-3 protein, relative to A549-wild type (WT) cells, was retained following stable transfection.

### Human Primary Epithelial Cells (HpEC)

HpEC of bronchial origin were maintained in bronchial epithelial basal medium (BEBM) supplemented, as directed (all purchased from Lonza, Walkersville, MD). Cultures were passed twice weekly with a portion of HpEC used to seed 96-well plates for co-culture experiments, as described above for A549-WT and clones. In general, experiments involving the use of HpEC were conducted within 10 passages, or until cells showed reduced capacity to proliferate. In preliminary experiments, BEBM was found to suppress cytokine production. For this reason, and to be consistent with the co-cultures using A549 EC, BEBM was carefully aspirated and replaced with 0.100 ml of F-12K nutrient mixture –Kaigh’s Modification, within 1h of performing co-cultures with DC.

### Coupling of Gal-3, Gal-9, and BSA to Microspheres

Polystyrene microspheres (MS) of 5μm size were purchased (Phosphorex, Inc. Hopkinton, MA) and washed twice with sterile ddH_2_0, according to manufacturer’s specifications. MS were then coated with BSA, Gal-3, or Gal-9 by using the same conditions commonly done when coating microtiter wells for ELISA. In brief, a saturating solution (10 μg/ml in PBS buffer, pH 7.2) was used for each protein. MS were incubated with solutions (0.5 ml per ~3x10^7^ MS) for 1h at room temperature (~23°C) with mixing every 15 min. before incubating overnight at 4°C. After coating, MS were centrifuged, supernatants removed, and then washed 3x with PBS, 0.1% BSA. MS were counted, adjusted to 1x10^8^/ml, and stored at 4°C. MS proved functional for several months when prepared/kept under sterile conditions. Importantly, it was later reasoned that the surfactant added to these MS, as supplied by the manufacturer, often showed signs of interference when coupling protein. As a result, later experiments switched to aldehyde sulfate polystyrene MS (Invitrogen/ThermoFisher, Grand Island, NY) of the same size (5μm), but supplied in a surfactant-free solution. These MS were similarly coated with protein, with the exception of using a 25mM MOPS buffer (pH 7.0) rather than PBS.

### Isolation of Monocytes and DC Subtypes From Blood

Monocytes and DC subtypes were prepared from residual TRIMA cassettes from anonymous subjects undergoing platelet pheresis. In some instances, venipuncture was performed on consenting adults (age range, 21–65 years) using a protocol approved by the Johns Hopkins University Institutional Review Board. Subjects were selected regardless of allergic status. Buffy-coats from both specimen sources were subjected to double-Percoll density centrifugation, which produces both basophil-depleted cell (BDC) and basophil-enriched cell (BEC) suspensions, as described (20). The BDC suspensions were washed 4x to remove platelets before preparing monocytes and DCs using positive selection with immunobeads on magnetic LS columns (Miltenyi Biotec, Gaithersburg, MD). Importantly, monocytes were first depleted from the BDC using CD14^+^ immunobeads and two passes over LS columns. And, those from the first pass were recovered from the columns, washed, counted, and used in some of the experiments. Then, pDC were prepared using BDCA-4^+^ (CD304) selection whereas Cd1c^+^ mDC were isolated using BDCA1^+^ selection, as previously described (3, 4) –both kits from Miltenyi Biotec, Gaithersburg, MD). Starting with 2.5x10^8^ BDC, enriched pDC and mDC suspensions averaged between 1–2x10^6^ and 2–3x10^6^, respectively. The few numbers of DCs isolated did not always allow for flow cytometric analysis, but previous studies indicate purities in the range of 50%–90%, based on CD123^+^BDCA2^+^ (pDC) and BDCA1^+^ (mDC) staining (3, 4).

Although CD14^+^ cells were depleted prior to isolating DC subtypes, flow cytometric analyses indicated residual monocytes (5%–20%) in some of the pDC suspensions. Since these contaminating monocytes likely contributed to the levels of TNF-α/IL-6 detected, later experiments additionally made use of pDC (and mDC) isolated by negative selection (StemCell Technologies, Vancouver, Canada). DC prepared in this manner showed no evidence of monocyte contamination, as assessed by flow staining for CD14^+^ cells.

### Culture-Derived DC (CDDC) and Passive Sensitization With IgE

CDDCs were prepared as previously described with slight modification (21). In brief, hematopoietic precursors were isolated from 1-2x10^9^ BDC using CD34^+^ positive selection (Miltenyi Biotec, Gaithersburg, MD). The number of CD34^+^ enriched cells ranged between 1-3x10^6^, which were then seeded at 1x10^5^/ml in serum-free StemPro-34 medium containing Flt-3 ligand (50 ng/ml), TPO (50 ng/ml), SCF (25 ng/ml), IL-3 (20 ng/ml) and SR-1 (0.5 mM). After 21–24 d at 37°C, 5% CO_2_, the cells, which had expanded in number by 30–35-fold, were harvested, washed 2x, counted, resuspended in C-IMDM and split 3-ways for passive sensitization accordingly: 1) medium alone, 2) with JK IgE (2μg/ml), and 3) with SE44 IgE (2μg/ml). These were cultured at 37°C, 5% CO_2_ for three additional days before testing, which included phenotyping, RT-PCR, and functional assays.

### Flow Cytometry

Surface Gal-3 protein was detected using flow cytometry, as previously described (9). The expression of various markers on DC subtypes isolated from blood and those expressed by CDDC were also assessed using flow cytometry. In all instances, blocking was done using 1 mg/ml human IgG. Staining for Gal-3 was achieved indirectly by first using monoclonal anti-Gal-3 antibody or an IgG_1_ isotype control (both at 10 μg/ml) for 20 min incubations at RT. Cells were then washed before staining 30 min on ice with goat anti-IgG_1_-Alex647 (2.5 μg/ml). All other staining was done directly using PE-labeled antibodies. Flow cytometry was performed using a FACSCalibur machine.

### Immunofluorescence (IF)

HpEC were seeded onto cell culture slides (MatTek Corp., Ashland, MA). After 48h, adherent cells were gently washed with pre-warmed (37°C) PBS, before fixing with buffered 4% paraformaldehyde (PF) for 5 min at R.T. (~23°C). Cell chambers were immediately flooded with PBS, 0.1% BSA. After aspirating wash fluid, cells were then blocked 1h using 1 mg/ml human IgG. The monoclonal anti-Gal-3 antibody and IgG_1_ isotype control were each used at 10 μg/ml. Slides were incubated 2h at RT before placing at 4°C for overnight. Cells were then washed 3 times with PBS, 0.1% BSA before adding anti-IgG_1_-Alex594 (1.0 μg/ml). After incubating 1 h at R.T., cells were washed 3 times. Chambers were then removed, the slide washed 1x with ddH_2_O, before mounting with medium containing DAPI (Millipore, Burlington, MA). IF was observed with a Nikon Eclipse Ti-U microscope equipped with DS-Fi2 camera (Nikon).

### Co-Culture Conditions

All cultures to induce cytokine production by the DC clones, monocytes, and CDDC were done, as described (9). In brief, cells were suspended in C-IMDM such that 1–2x10^4^ were added in 0.050 ml volumes to flat-bottom wells (96-well plates) pre-plated with EC (A549-WT, clones, HpEC, or medium alone –all at 0.100 ml). Likewise, the same volume and cell numbers were added to round-bottom wells containing 0.100 ml vol. of 0.5–1.0x10^5^ MS or medium alone. Immediately after adding DCs, monocytes, or CDDC, 0.050 ml of 4x stimulus (e.g., IL-3, anti-IgE, or medium alone) was added and the cultures incubated as indicated at 37°C, 5% CO_2_. In some instances, soluble Gal-3 was added in place of EC and MS. Supernatants were harvested after 20h unless otherwise indicated and tested for cytokine secretion.

### Real-Time RT-PCR

Total RNA was isolated using the Trizol protocol (Tel-test, Inc, Friendswood, Tex). After isopropanol precipitation, RNA was washed with ethanol before resuspending in DNase-free water. This RNA was then subjected to the “RNA cleanup” protocol (Qiagen Corp., Germantown, MD) and quantified using Nanodrop. Quantitative RT-PCR was performed using validated primer/probe combinations for FcεRIα (Applied Biosystems, Foster City, CA).

### Cytokine Measurements

Supernatants were analyzed for TNF-α and IL-6 protein by ELISA (ThermoFisher. Grand Island, NY).

### Statistical Analysis

Statistical analyses were performed with Prism 7.0 software (GraphPad, Software, LaJolla, Calif.) Analyses were performed using Wilcoxon non-parametric and/or paired t-test analyses unless otherwise specified. Differences were considered statistically significant at a *P* value <0.05.

## Results

### Gal-3 Activates Human Blood DC for Pro-Inflammatory Cytokines: Matrix-Bound vs. Solution

**Figure 1:**
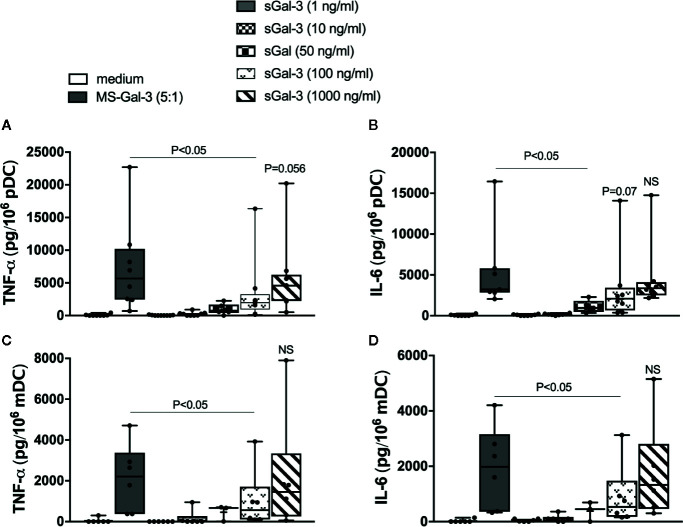
Gal-3 activates human blood dendritic cell (DC) subtypes for pro-inflammatory cytokines: matrix-bound vs. solution. rhGal-3 was passively absorbed onto 5μm-sized microspheres (MS-Gal-3), as described in the *Material and Methods* section, or directly added to culture medium (sGal-3) at the indicated concentrations. Human plasmacytoid DCs (pDCs) **(A, B)** and myeloid DC (mDC) **(C, D)** were then co-cultured with MS-Gal-3 at a 5:1 ratio (MS:DC) or directly added to increasing concentrations of sGal-3 or medium alone. Cell culture supernatants were then harvested after 20h incubation and assayed for TNF-α and IL-6 by ELISA. Results are shown in Box-Whisker plots representing results from different DC preparations (n=3–8).

We began by testing Gal-3 for its capacity to induce TNF-α and IL-6 from pDC and mDC isolated from blood. However, we had previously shown that Gal-3, when used in solution, did not readily activate basophils compared to when it is coupled to a solid matrix (10). Therefore, we compared whether DCs are likewise activated by microspheres (MS) conjugated with Gal-3 (MS-Gal-3) vs. using this lectin in solution (sGal-3). As shown in **Figure 1**, sGal-3 activated both pDC and mDC for TNF-α and IL-6 but only at concentrations ≥50 ng/ml, which far exceed the levels commonly reported in plasma, even during disease states reporting the high levels of this lectin (22). For comparison, MS-Gal-3 activated both DC subtypes to produce more of each cytokine, either significantly or with a strong trend, than those induced using sGal-3 at 100 ng/ml. Comparable activity, in fact, was only observed when sGal-3 was used at the highest concentration tested, 1,000 ng/ml.

### Time Course for Gal-3-Dependent Induction of TNF-*α* and IL-6 From DC Subtypes

**Figure 2:**
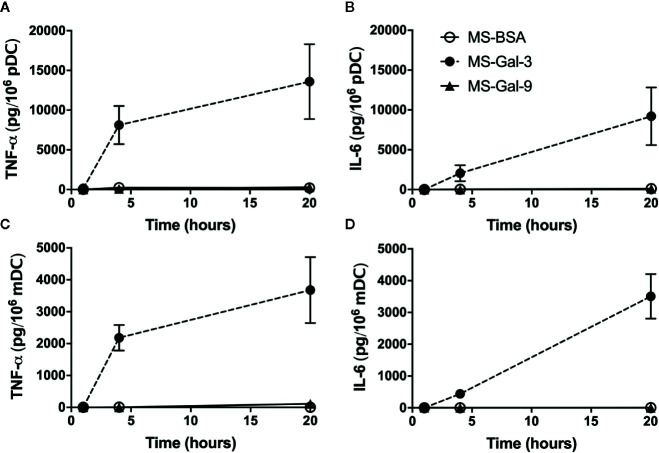
Time course for Gal-3-dependent activation of TNF-α and IL-6 from dendritic cells (DCs). Human plasmacytoid DC (pDC) **(A, B)** and myeloid DC (mDC) **(C, D)** were co-cultured with MS-Gal-3, MS-BSA, or MS-Gal-9, all at a 5:1 ratio (MS:DC). Supernatants were harvested at the indicated times and assayed for TNF-α and IL-6 protein. Values are the Mean ± SEM, n = 3.

In observing that matrix-bound Gal-3 robustly activated DC, much like we had previously shown for basophils, additional parameters were explored using the MS approach. Included in these experiments were conditions involving the use of MS coated with BSA (MS-BSA) and Gal-9 (MS-Gal-9) to help assess the specificity of the MS-Gal-3-dependent DC responses. Gal-9 was chosen because it too is reported to bind IgE with ~100-fold greater affinity, yet had shown no capacity to activate basophils (10). Notably, it was observed during these experiments that both MS-Gal-3 and MS-Gal-9 were qualitatively more adhesive, as evidenced by their tendency to stick to the tubes used to store stock suspensions (data not shown). The same was not observed for MS-BSA. We therefore investigated other parameters by looking at the time course for the TNF-α and IL-6 induced by MS-Gal-3. As shown in **Figure 2**, marked levels of TNF-α and IL-6 were again seen from both pDC and mDC co-cultured with MS-Gal-3. In fact, the time courses for both responses showed remarkably similarity to those previously reported for these DC subtypes following IgE/FcεRI-dependent activation (2, 4). In particular, TNF-α levels after 4 h incubation were nearly 60%–70% of those produced after 20 h. For comparison, the IL-6 produced at 4 h was just ~15%–20% of that secreted after 20 h. Importantly, both MS-BSA and MS-Gal-9 showed no capacity to activate either DC subtype with regard to TNF-α and IL-6 production.

### The Effect of Co-Stimulation With IL-3 and Anti-IgE on Gal-3-Dependent Activation of DC Subtypes

**Figure 3:**
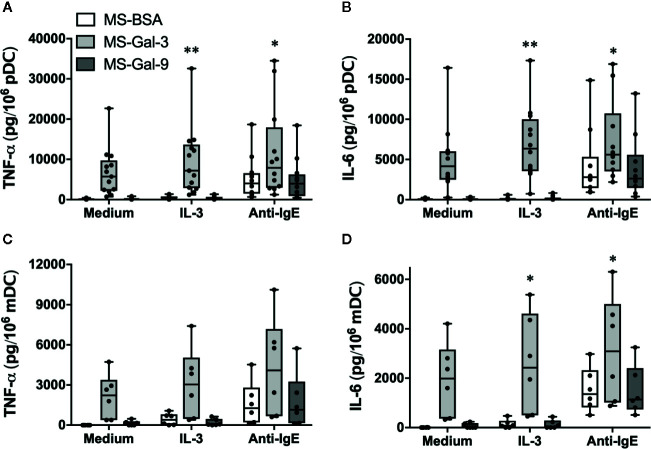
The effect of co-stimulation on Gal-3-dependent activation of dendritic cells (DCs). Human plasmacytoid DC (pDC) **(A, B)** and myeloid DC (mDC) **(C, D)** were co-cultured with MS-Gal-3, MS-BSA, or MS-Gal-9 as described above, but additionally in the presence of IL-3 (10 ng/ml), Anti-IgE (5μg/ml), or medium alone. Supernatants were harvested after 20 h and assayed for TNF-α and IL-6. Individual values (n=6-12) are shown within Box-Whisker plots. Co-stimulation with IL-3 and Anti-IgE were investigated for significance vs. using MS-Gal-3 alone. **P ≤ 0.008, *P < 0.05.

We had previously shown that IL-3 greatly facilitated Gal-3-dependent activation of basophils for the production of IL-4/IL-13. Since pDC, like basophils, express high levels of IL-3 receptor (CD123), we next tested whether the addition of IL-3 mediated co-stimulatory activity when DCs are co-cultured with MS-Gal-3. For this series of experiments, we also explored the effects of co-stimulation with anti-IgE, which is known to stimulate DC to produce TNF-α and IL-6. As shown in **Figure 3**, MS-Gal-3 once again activated both pDC and mDC to secrete abundant amounts of TNF-α and IL-6, whereas neither MS-BSA nor MS-Gal-9 activated either DC subtype to produce these cytokines, even with IL-3 co-stimulation. While we observed that the addition of IL-3 significantly augmented the MS-Gal-3-dependent responses by pDC (p ≤ 0.008), and trended to do so for mDC (p ≤ 0.07), the overall effects were minimal, amounting to ~40-60% increases above the levels induced by MS-Gal-3 alone. Importantly, both DC subtypes responded to anti-IgE, as evident by comparable levels of TNF-α and IL-6 produced with this stimulus alone, and after it was added to co-cultures also containing MS-BSA or MS-Gal-9. Interestingly, the levels of cytokine secreted by DCs co-stimulated with anti-IgE and MS-Gal-3 appeared additive and were greater than those using either stimulus alone. Importantly, the addition of goat IgG (used as an isotype control for the anti-IgE) did not mediate DC activation (data not shown), which is consistent with our previous reports (2, 4).

### Gal-3-Dependent Induction of DC-Associated Activation Markers

**Figure 4:**
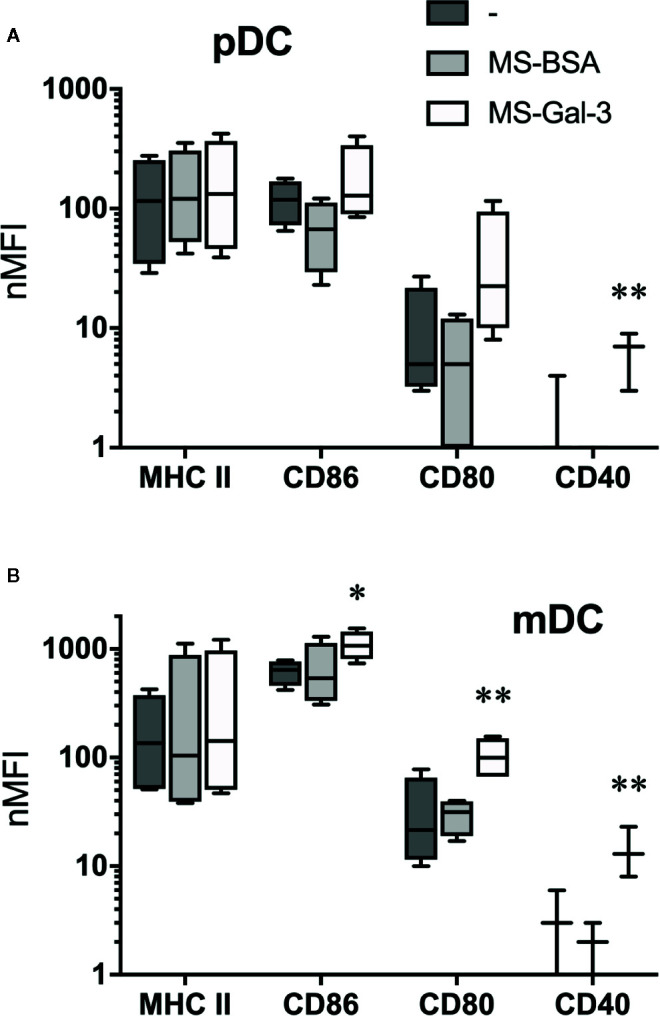
Gal-3-dependent induction of dendritic cell (DC) activation markers. **(A)** Plasmacytoid DC (pDC) and **(B)** myeloid DC (mDC) were co-cultured with MS-Gal-3, MS-BSA, or medium alone. After 20 h incubation, cells were analyzed by flow cytometry for expression of the indicated markers. Shown are Box-Whisker plots, n = 3-4. **P < 0.02 and *P < 0.05 vs. medium alone.

To substantiate the notion that Gal-3 activates pDC and mDC, we also investigated changes in the expression of DC activation markers after co-incubating 20 h with MS-Gal-3, MS-BSA, or medium alone. As shown in **Figure 4**, the expression of CD80, CD86, and CD40 significantly (p ≤ 0.05) increased on mDC co-cultured with MS-Gal-3. Similar trends were also observed for pDC, but only CD40 was significantly increased for this DC subtype.

### Gal-3 Expression Is Essential for A549 Epithelial Cell-Dependent Activation of Monocytes and DCs

**Figure 5:**
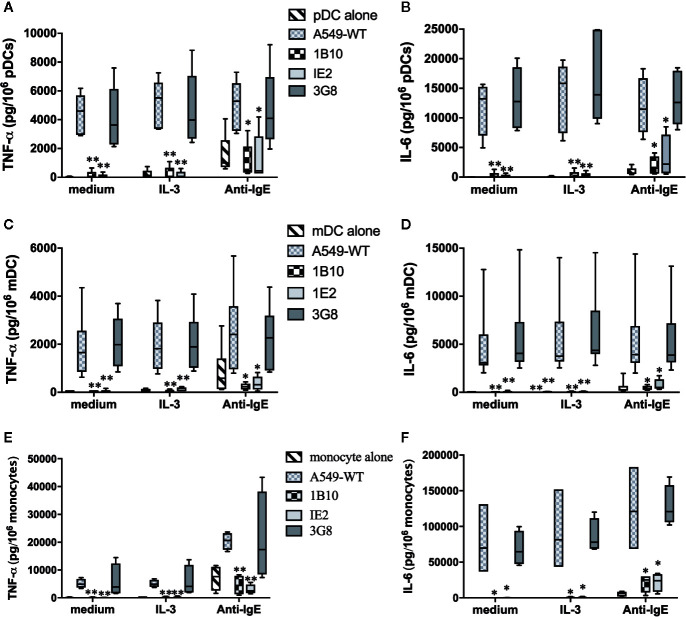
Activation of dendritic cells (DCs) and monocytes by A549 EC for pro-inflammatory cytokines is dependent on EC-associated Gal-3. Plasmacytoid DC (pDC) (**A, B**, n=5), myeloid DC (mDC) (**C, D**, n=8), and monocytes (**E, F**, n=4) were co-cultured with A549-WT, Gal-3-deficient clones (1B10 & 1E2), and Gal-3^+^ clone, 3G8, along with IL-3 (10 ng/ml), anti-IgE (5 μg/ml), or medium alone. Supernatants were harvested after 20h incubation and assayed for TNF-α and IL-6. *P ≤ 0.05; **P ≤ 0.001 vs. corresponding conditions with DC+A549-WT.

To further support the relevance of Gal-3-dependent activation of DC, we investigated whether pDC and mDC are similarly activated when co-cultured with A549 ECs. Again, experiments utilizing this cell line proved instrumental in demonstrating that Gal-3 activates human basophils for histamine release and IL-4/IL-13 secretion (9, 10). We therefore explored whether A549 EC would mimic the DC responses seen using MS-Gal-3. And, since monocytes are reported to respond to IgE-dependent stimulation, we additionally investigated whether these cells would similarly respond to A549 EC, knowing that they also secrete high levels of TNF-α/IL-6. As shown in **Figure 5**, pDC, mDC, and monocytes were all activated to secrete TNF-α and IL-6 when co-cultured with wild-type A549 (A549-WT). For the DCs, the levels detected were comparable to those induced by Gal-3-MS. Importantly, two lines of evidence indicated that these cytokines were, indeed, produced by the DCs/monocytes and not by the EC line. First, TNF-α and IL-6 were not detected in supernatants from A549-WT cultured without DC. Moreover, A549-WT, if made incapable of secreting cytokine by first fixing with paraformaldehyde, retained the capacity to activate both DC subtypes (**Figure S2**). In sharp contrast, A549 clones 1B10 and 1E2, for which Gal-3 expression is repressed >95% (10), showed essentially no capacity to activate pDC, mDC, or monocytes for TNF-α and IL-6. Yet, a control clone, 3G8, which expresses ~50% of the Gal-3 seen in A549-WT cells (10), retained the capacity to activate all three cell types, although generally with less stimulatory activity. In addition, a nearly identical pattern (as to that seen using MS-Gal-3) was observed if A549/DC co-cultures also received IL-3, which only modestly augmented DC stimulation mediated by A549-WT. As expected, both DC subtypes and monocytes responded to stimulation with anti-IgE, albeit secreting significantly less TNF-α and IL-6 than when co-cultured with A549-WT. Importantly, these responses to anti-IgE were retained even when all cell types were co-cultured with the Gal-3-deficient clones.

Since DC subtypes were isolated using positive selection, which might conceivably impact the overall responses observed, we conducted several A549 co-culture experiments using pDC and mDC isolated by negative selection. This approach also eliminated residual CD14^+^ that were often detected in the pDC preparations (presumably monocytes co-expressing BDCA4) and which potentially contributed to the overall cytokine production. Consequently, cytokine levels (e.g., IL-6) were generally less in co-cultures using negatively selected pDC, but still readily detected in the ng/10^6^ range (**Figure S3**).

### Human Primary Epithelial Cells Expressing Gal-3 Activate DC for Pro-Inflammatory Cytokines

**Figure 6:**
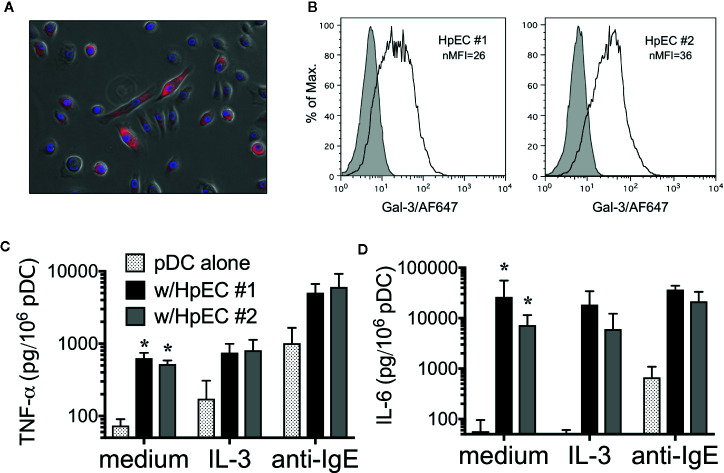
Human primary epithelial cells (HpEC) express Gal-3 and activate plasmacytoid dendritic cell (pDC). Staining of two different HpEC specimens (bronchial-derived) for Gal-3 protein using: **(A)** immunofluorescence (IF) and **(B)** Flow cytometry. **(C)** TNF-α and **(D)** IL-6 production by pDC co-cultured with HpEC #1 and #2, as indicated. Values shown for co-cultures in medium alone are the Mean ± SEM, n=5. *P < 0.003. Values for co-cultures in the presence of IL-3 (10 ng/ml) and anti-IgE (5μg/ml) are the Mean ± SEM, n = 2.

Since A549 is an adenocarcinoma cell line and thus not truly representative of normal EC, experiments were also done to explore whether human primary epithelial cells (HpEC) mediate a similar capacity to activate DC. First, two different specimens (both from bronchial tissue) were found to express Gal-3 protein, as determined by IF (**Figure 6A**) and by flow cytometry (**Figure 6B**). More importantly, both HpEC specimens also activated pDCs (isolated from several different donors) to secrete TNF-α (**Figure 6C**) and IL-6 (**Figure 6D**) and in a manner nearly identical to what had been observed when co-culturing pDC with A549-WT cells (and MS-Gal-3). Once again, neither TNF-α nor IL-6 was detected in supernatant from HpEC cultured alone (data not shown), indicating that they were, indeed, pDC-derived.

### Gal-3 Dependent Activation of DC Occurs Independently of IgE Expression

**Figure 7:**
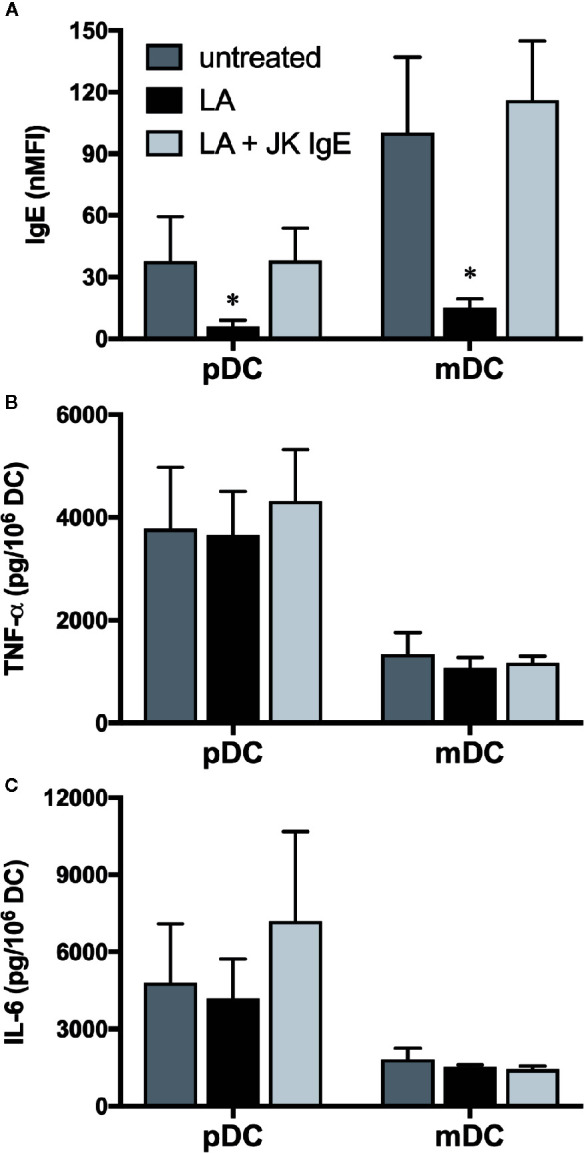
Dendritic cell (DC) responsiveness to Gal-3-dependent activation is not altered by removal of IgE. **(A)** IgE expression on untreated pDC and mDC or following lactic acid (LA) treatment ± passive sensitization with JK-IgE (2 μg/ml). **(B)** TNF-α and **(C)**, IL-6 protein levels secreted from a portion of the cells treated in **(A)** after co-culturing with A549-WT for 20h. Results are the Mean ± SEM, n=3. *P<0.05.

As noted, the Gal-3-dependent responses observed with human basophils proved dependent on these granulocytes expressing IgE (10) and required FcεRI-dependent signaling (9). Because human pDC and mDC also bind IgE, albeit *via* the so-called αγ_2_ variant of FcεRI, we next explored whether the Gal-3-dependent activation observed for these cells is likewise dependent on IgE by utilizing approaches similar to those done for basophils. The first approach involved “stripping” of IgE using lactic acid buffer, pH 4.0, which has long been employed for removing IgE from basophils. Indeed, staining for endogenous IgE on basophils treated just 1 min. with this buffer decreased by ~80% (data not shown). As shown in **Figure 7** the same treatment also decreased IgE staining on pDC and mDC by >80%. Yet, IgE expression on both DC subtypes was restored to ≥85% by re-sensitizing a portion of the “stripped” cells. Regardless, there were essentially no changes in DC responsiveness (i.e., TNF-α & IL-6 secretion) to A549-dependent activation, as a result of removing IgE, either from pDC or mDC.

**Figure 8:**
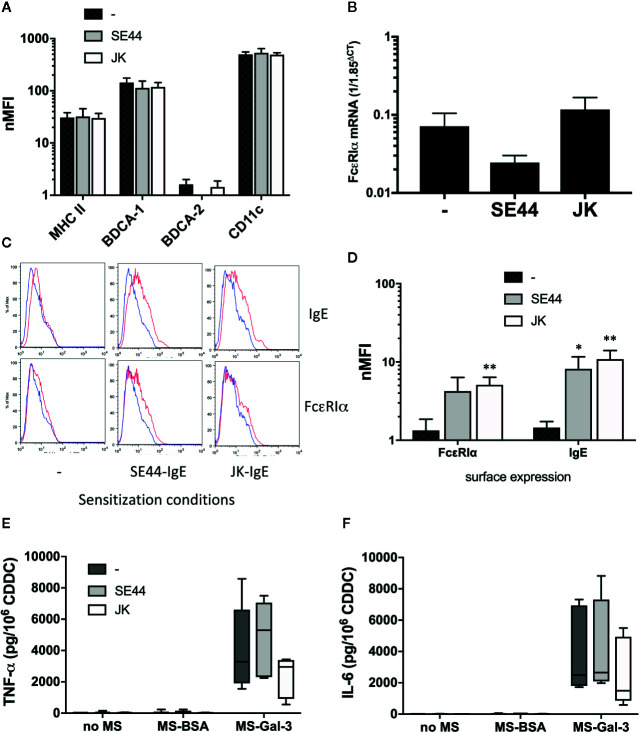
IgE expression by culture-derived dendritic cell (CDDC) is not required for Gal-3-dependent activation. CDDC were grown from CD34^+^ precursors, as described in the *Materials & Methods* section. CDDC were then portioned, with one-third passively sensitized with JK-IgE, one-third with SE44-IgE (both at 2 μg/ml), and one-third left untreated. After 3d incubation, cells from all three conditions were washed and analyzed for the following: **(A)** phenotypic markers, **(B)** mRNA expression for FcεRIα, **(C, D),** expression of FcεRIα protein & IgE, and **(E, F)**, induction of TNF-α and IL-6, respectively, after 20 h co-culture with MS-BSA, MS-Gal-3, or no MS. All bar graphs are the Mean ± SEM, n = 4. *P<0.05, **P<0.01.

Although low pH treatment removed ~80% of the surface IgE endogenous to blood pDC and mDC, it remained possible that the ~20% not removed might still be sufficient to allow Gal-3-dependent activation. We therefore employed the approach of growing DC from CD34^+^ precursors (21), but in serum-free medium lacking IgE. This would allow tests to compare phenotypic and functional differences, whereby a portion of the culture-derived DCs (CDDC) could be sensitized with IgE and the remaining cells left un-sensitized. First, an analysis of markers showed that CDDC were MHC II^+^CD11c^+^BDCA-1^+^BDCA4^-^, which is consistent with an mDC phenotype (**Figure S1**). As shown in **Figure 8**, every CDDC preparation showed this phenotype, which was not altered by passive sensitization using two different IgEs. Importantly, CDDC also showed baseline mRNA expression for FcεRIα. And, as expected, sensitization with IgE resulted in a significant increase of FcεRIα protein on the surface of CDDC, which then allowed for greater detection of IgE, presumably *via* more binding of the immunoglobulin. However, CDDC secreted comparable levels of TNF-α and IL-6 in response to Gal-3-dependent stimulation, regardless of whether they were sensitized with IgE.

## Discussion

In exploring mechanisms whereby epithelial cells (EC) might activate human basophils, we serendipitously discovered a role for Gal-3 in stimulating these granulocytes for mediator release and cytokine secretion (9). Importantly, this mode of activation, which we demonstrated first by using the A549 EC line, proved independent of EC-derived soluble factors such as cytokines (e.g., TSLP, IL-33), but was instead dependent on cell-to-cell contact. Moreover, basophil responsiveness in these co-cultures was fully dependent on their expression of IgE and FcεRI-dependent signaling. Consequently, we then showed that the ability of A549 to activate basophils was fully dependent on these EC expressing Gal-3, since knocking-down the expression of this lectin completely abrogated the capacity of A549 clones to stimulate basophils (10). Interestingly, attempts to use rhGal-3 showed that it barely activated basophils and did so only when used at concentrations (e.g., 10 μg/ml) that far exceed the plasma levels reported in various pathological conditions (22). However, the ability of rhGal-3 to inhibit A549-dependent activation of basophils (likely by competing with Gal-3 anchored on A549 EC) gave indication that it was, indeed, active. Hence, we then demonstrated that microspheres (5 μm in size) coated with rhGal-3 (but not BSA or Gal-9) activated basophils in a manner similar to that seen with A549 EC, thus indicating that this lectin, when immobilized, may better facilitate immune cell activation (10).

For the very reason that Gal-3 was initially described as an IgE-binding lectin more than 30 years ago (11) and now more recently shown to activate basophils in an IgE-dependent manner, we addressed herein whether other IgE bearing cells are likewise affected by this lectin. Our rationale for testing human blood DC subtypes (pDC & mDC) and monocytes stemmed from the substantial amount of evidence that these cells also bind IgE with high affinity, albeit through a variant (αγ_2_) of FcεRI. Moreover, these cells secrete high levels of TNF-α and IL-6 when stimulated with antibodies capable of cross-linking IgE (2, 3, 6) or FcεRIα (4). And, in the case of pDC, which share with basophils the high expression of IL-3 receptor (CD123), we considered co-stimulation with this cytokine might be required, as it proved critical in facilitating basophil activation to Gal-3.

Accordingly, we began by investigating DC responsiveness to Gal-3 by co-culturing these cells with microspheres pre-absorbed with rhGal-3 (MS-Gal-3) in a manner much like we had done with basophils. Upon doing so, we discovered that both pDC and mDC responded by secreting relatively high levels (ng/10^6^ cells) of TNF-α and IL-6. Whereas co-stimulation with IL-3 was required for MS-Gal-3 to induce IL-4 and IL-13 from basophils, it was not necessary for inducing cytokine production by either DC subtype, including pDC. Nonetheless, IL-3 did significantly augment the MS-Gal-3-dependent responses of pDC by ~40%–60%, which again, was expected given the high-level expression of CD123 on these cells and the fact they are often responsive to this cytokine. As was observed in the basophil studies, both pDC and mDC were only responsive to MS-Gal-3 and did not react to either MS-BSA or MS-Gal-9. We initially concluded that these observations are likely relevant to the same differences we had witnessed between Gal-3 and Gal-9 in the activation of basophils (10). In particular, Gal-9, despite binding IgE with a reported ~100-fold greater affinity (14), has a tandem repeat structure, which is only conducive to bivalent binding. With this structure, Gal-9 is perhaps unable to crosslink multiple IgE/FcεRI complexes, which is typically necessary for activation. In contrast, Gal-3’s pentamer structure does allow for the binding of multiple IgE molecules and hence the theoretical activation of basophils (and perhaps DC) *via* crosslinking of IgE/FcεRI complexes. Obviously, additional studies are necessary to determine whether these structural differences actually account for the activating capacity of Gal-3 vs. Gal-9.

Just as Gal-3 demonstrated a greater capacity to activate basophils when absorbed onto MS than when used in solution (sGal-3), the same proved true for the activation of DC by this lectin. For example, TNF-α and IL-6 secretion by pDC and mDC were not consistently induced with sGal-3 until a concentration of 50 ng/ml was achieved, and even then, this only amounted to 10%–20% of those seen using MS-Gal-3. In fact, only when used in at the highest concentration tested, 1,000 ng/ml, did sGal-3 achieve the same level of activation observed with the microspheres. Certainly, it remains unclear at this time why matrix-associated Gal-3, (whether it be MS or EC) stimulates DCs better than if in solution, but it likely relates to the belief that immobilized lectin is better able to crosslink activation-linked receptors (e.g., FcεRI). Nonetheless, this phenomenon may have clinical implications as well. For instance, increased concentrations of Gal-3 in plasma/serum have been reported and, in some instances, are indicative of disease (e.g., cardiovascular disease) (22). Yet, these concentrations typically do not exceed those (~50 ng/ml) that are seemingly necessary to activate blood DCs (and basophils). In contrast, it has recently been reported that Gal-3 concentrations can exceed 50 ng/ml (up to 500 ng/ml) in the draining lymph nodes (DLN) of subjects with metastatic melanoma (23). Thus, it is reasonable to hypothesize that DCs infiltrating the tumor microenvironment (TME) could be stimulated to secrete TNF-α and IL-6, and are more likely to do so if they were to come in contact with EC (or tumor cells) expressing Gal-3. In this regard, the Broggi, et al. study also reported both TNF-α and IL-6 as prominent cytokines in the same DLN also showing high Gal-3 levels (23). Thus, the above hypothesis that a Gal-3-rich TME (which, in fact, is common to various cancers) functions in stimulating local and/or infiltrating DC to secrete these cytokines, is a real possibility. Moreover, chronic levels of TNF-α and IL-6 in the TME have been linked to such phenomena as T cell exhaustion and facilitated tumor growth, respectively (24). Indeed, there is rationale for Gal-3 acting as a checkpoint inhibitor (25). Perhaps its ability to do so is mediated, in part, by its capacity to stimulate chronically high levels of TNF-α and IL-6 from DC.

The pattern of DC responsiveness to EC expressing Gal-3 was identical to, if not more potent than, that observed using MS-Gal-3. In fact, both pDC and mDC suspensions secreted copious amounts of TNF-α and IL-6 when co-cultured with A549 EC or HpEC. And, like those responses observed when DCs were co-cultured with MS-Gal-3, no co-stimulation was required. Both DC subtypes secreted these cytokines without the need for IL-3 or anti-IgE. The best evidence that this response was, indeed, dependent on the Gal-3 expressed by A549-WT was demonstrated by the near lack of responsiveness when either DC subtype was co-cultured with clones (IE2 & 1B10) deficient in Gal-3 protein. Yet, the TNF-α and IL-6 responses persisted when pDC and mDC were co-cultured with clone 3G8, which had been previously shown to retain Gal-3 protein at ~50% of that express by A549-WT. Likewise, clone 3G8 had also retained the capacity to activate basophils (10). Importantly, both DC subtypes responded to anti-IgE antibody, even when co-cultured with the Gal-3-deficient clones, signifying a functional capacity to secrete TNF-α and IL-6 to a different mode of stimulation. And, quite similar cytokine responses (and levels) were observed when pDC and mDC were co-cultured with HpEC, indicating that such responses are not limited to the A549 cell line. Whether these HpEC responses are also dependent on Gal-3 was not determined in this study, but the pattern and extent of responsiveness would strongly indicate this possibility. Forthcoming studies will definitively address the dependency of Gal-3 in this reaction, as well as the parameters regulating the expression of this lectin on HpEC.

We further discovered that monocytes likewise produced ample amounts of TNF-α and IL-6 when co-cultured with A549 EC (including the 3G8 clone), yet failed to secrete these cytokines when co-cultured with the Gal-3-deficient clones. Indeed, these responses were not unexpected knowing that monocytes also express FcεRI (the αγ_2_ variant) and, assuming an underlying mechanism involving Gal-3 binding to IgE, could be triggered to secrete TNF-α/IL-6. Of course, in light of the cytokine levels produced by these cells, we soon reasoned that at least a portion of the cytokines produced by pDC isolated by BDCA4^+^ selection was derived from residual monocytes contaminating these suspensions. Hence, supplementary experiments were done to show that pDC (and mDC) isolated by negative selection (which showed no monocyte contamination), retained the capacity to respond to A549 EC –findings that proved positive and thus strengthen the overall concept.

When investigating A549/Gal-3-dependent activation of human basophils for IL-4 and IL-13, we clearly demonstrated that IgE expression was important in this response (9). Naturally, we hypothesized herein that blood DC (both pDC and mDC) would also need to express IgE in order to be responsive to A549/Gal-3-dependent activation. However, we used two different approaches to test the requirement for DC-associated IgE and both proved negative. In particular, we demonstrated that nearly 80% of the endogenous IgE expressed by DC could be removed using “lactic acid stripping”, yet the cells retained the capacity to respond to Gal-3-dependent activation. Certainly, this low-pH approach has long been employed to remove IgE from basophils (26). And, we had previously used lactic acid to demonstrate that basophils require IgE expression in order to react to A549-WT (9). In contrast, we show herein that both pDC and mDC, despite being stripped of >80% of surface IgE, retained the capacity to secrete TNF-α and IL-6 when co-cultured with A549-WT, and at levels comparable to control cultures in which DCs were not treated with lactic acid. Obviously, it remained possible that the ~20% of endogenous IgE that was not removed with lactic acid, might still allow DCs to respond to Gal-3-dependent activation. We therefore proceeded by growing DC from CD34^+^ precursors in serum-fee medium lacking IgE. These cells expressed markers (e.g., BDCA1/CD1c^+^BDCA2^-^) consistent with an mDC-like phenotype. Importantly, they also expressed FcεRIα, making it possible to sensitize with exogenous IgE. Yet, when we achieved doing this, the binding of IgE did not alter their ability to secrete TNF-α/IL-6 in response to Gal-3-dependent activation. In fact, even CDDC never exposed to IgE responded as well as those sensitized for 3d with this immunoglobulin. We therefore conclude that unlike basophils, blood DCs (both pDC and mDC) do not require IgE expression in order to respond to Gal-3-dependent activation.

With our data clearly showing that IgE expression by DCs is dispensable for responsiveness to Gal-3-dependent activation, it remains unknown as to what glycoprotein(s) on pDC and mDC (and monocytes) this lectin interacts with to induce TNF-α and IL-6. As it stands, there is a growing list of possibilities, which will likely require empirical testing to determine exactly what protein(s) is responsible. In particular, FcεRIα remains a potential possibility that was not directly tested in our experiments. In fact, early studies showed that Gal-3 also bound to FcεRIα, which, like IgE itself, is heavily glycosylated (13). Certainly, antibodies targeting FcεRα are also reported to stimulate TNF-α and IL-6 from pDC & mDC with essentially the same parameters (4). Therefore, it remains possible that Gal-3 induces these cytokines *via* FcεRI-dependent activation, even without the expression of IgE. Unfortunately, the intracellular signaling pathways regulating FcεRI-dependent activation in DC remain poorly understood, thus making it difficult to address this issue pharmacologically. Approaches beyond the scope of this manuscript will be required to determine exactly what glycoprotein Gal-3 interacts with to induce DC activation.

Finally, Gal-3 has been linked to the activation of several immune cell types, including mouse and human monocyte-derived DC. However, these previous studies point mostly to the importance of endogenous or intracellular Gal-3 in regulating the secretion of pro-inflammatory cytokines by DC. We believe our data are the first to illustrate another way by which this lectin is capable of activating human immune cells for robust production of pro-inflammatory cytokines (i.e., TNF-α/IL-6). Specifically, these data point to how matrix cells expressing Gal-3 (e.g., EC) facilitate this stimulation by perhaps serving to anchor this lectin, thus allowing it to additionally bind/crosslink glycoproteins on DC (*via* open CRDs) to mediate activation. Indeed, Gal-3 is thought to promote “lattice” formation by binding cell surface glycoproteins that may also facilitate activation (12). The latter is of particular importance given that DC-EC axes are evident in various conditions/diseases. For example, it is well known that EC-derived TSLP promotes OX40L expression on DC to promote Th2 responses, including those underlying allergic disease. It is therefore not unreasonable to propose that EC-associated Gal-3 may similarly impact DC function by stimulating pro-inflammatory cytokine production in conditions where this galectin is highly expressed. Indeed, the significance of these data currently rest with the fact that Gal-3 is now identified as a biomarker and/or factor associated with the inflammation seen in a wide range of diseases/conditions, including various cancers, cardiovascular disease, autoimmunity (lupus), wound healing, and more recently, allergic disease, including asthma and atopic dermatitis (18). Certainly, the data herein reveal how EC-associated Gal-3 may very well be a vital component in the pathogenesis of these conditions by directly activating DC for pro-inflammatory cytokines, which, themselves, are often hallmark in many of the above conditions. Future studies should investigate this possibility, as they may further rationalize targeting Gal-3 therapeutically, and/or the glycoproteins binding by this lectin.

## Data Availability Statement

The datasets generated for this study are available on request to the corresponding author.

## Ethics Statement

The studies involving human participants were reviewed and approved by IRB, Johns Hopkins University. The patients/participants provided their written informed consent to participate in this study.

## Author Contributions

JS conceived the study, helped conduct experiments, and wrote the manuscript; AB and AA provided input regarding experimental design and conducted many of the experiments. All authors contributed to the article and approved the submitted version.

## Funding

Supported, in part, by Public Health Services Research Grants R01AI115703, R21AI121766, and R01 AI141486 to JS from the National Institute of Allergy and Infectious Diseases, National Institutes of Health (NIAID, NIH).

## Conflict of Interest

The authors declare that the research was conducted in the absence of any commercial or financial relationships that could be construed as a potential conflict of interest.
