# Supplementary material for: Epithelial Cell-Associated Galectin-3 Activates Human Dendritic Cell Subtypes for Pro-Inflammatory Cytokines
Source: Front Immunol. 2020 Oct 14;11:524826. doi: 10.3389/fimmu.2020.524826 (PMC7591743; doi:10.3389/fimmu.2020.524826)
Supplement: Supplementary file 1 [file DataSheet_1.pdf]

Supplemental Material

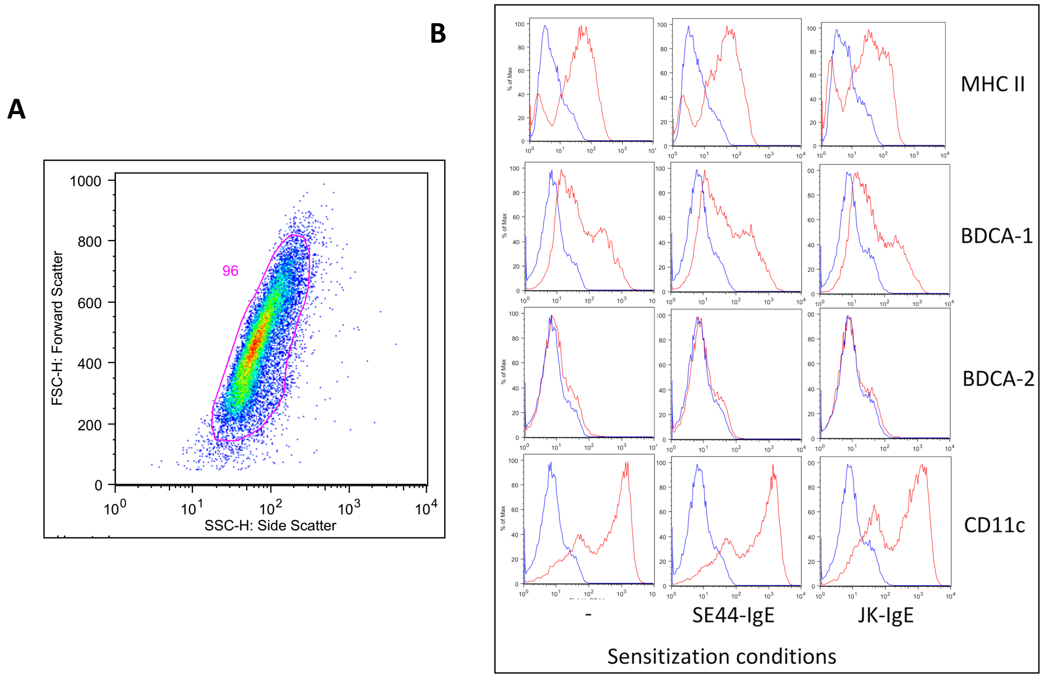

Figure S1. CDDC express markers consistent with an mDC phenotype. CD34^{+} cells were isolated and cultured as described in the *Materials and Methods* to obtain CDDC. After d21, CDDC were harvested, washed, and put back into culture in medium alone, with SE44-IgE (2µg/ml), or with JK-IgE (2µg/ml). After allowing 72h for sensitization, cells from all three conditions were harvested, washed, and analyzed by flow cytometry for expression of the indicated phenotypic markers. Results are from a representative experiment showing: ***A***, gating strategy and ***B***, staining for markers (red line) vs. isotype control (blue line).
