# Supplementary material for: Epithelial Cell-Associated Galectin-3 Activates Human Dendritic Cell Subtypes for Pro-Inflammatory Cytokines
Source: Front Immunol. 2020 Oct 14;11:524826. doi: 10.3389/fimmu.2020.524826 (PMC7591743; doi:10.3389/fimmu.2020.524826)
Supplement: Supplementary file 2 [file DataSheet_2.pdf]

Supplemental Material

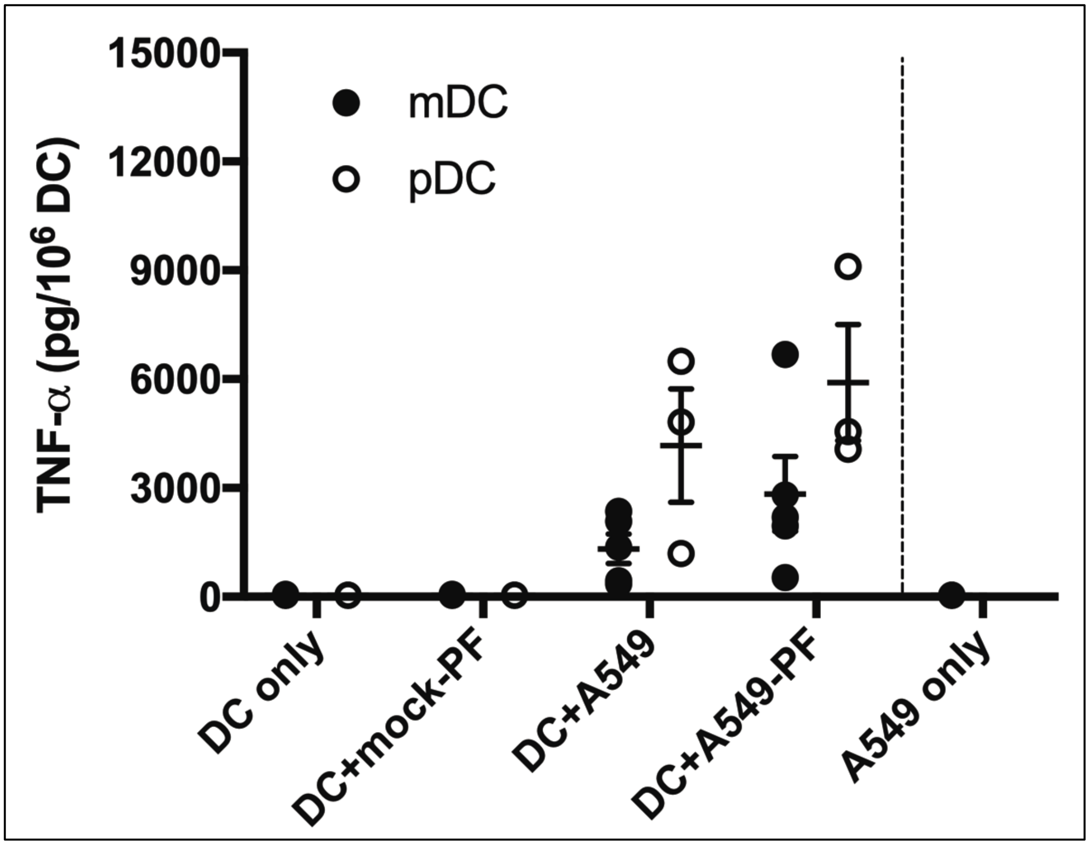

Figure S2. Paraformaldehyde (PF)-fixed A549 activate human DC subtypes for cytokine production. A549 cells were seeded into wells of 24-well plates and cultured 24h-48h to achieve 70-100% confluency. Selected cultures were then fixed with 4% buffered PF using the following approach: 1) culture supernatant removed by aspiration, 2) A549 monolayer washed with 1 ml pre-warmed (37˚C) PBS and then aspirated, 3) addition of 0.3 ml of 4% PF for 5 minutes at RT (~23˚C) before addition of 1 ml PBS 0.1% BSA, 4) well supernatants were then aspirated and the PBS 0.1% BSA wash repeated 2x more before adding fresh culture medium. A few wells without A549 were treated exactly the same way and used as "mock" controls for the PF fixing procedure, as indicated. After equilibrating all cultures (some with medium alone) to 37˚C, 5% CO_2 for 15 minutes, an equal vol. (0.250 ml) of DC ($5x10^4$) or medium alone was added as indicated and incubated for 20h. Supernatants were then harvested and assayed for TNF-α protein by ELISA. Error bars represent the Mean±SEM of the individual experiments (dots) conducted using cells prepare from different specimens (n=5 for mDC and n=3 for pDC). Results indicate that A549, even when fixed by PF, retain the capacity to activate both pDC and mDC and that the TNF-α produced is, indeed, derived from the DC subtypes and not from A549 cells.
