# Supplementary material for: Epithelial Cell-Associated Galectin-3 Activates Human Dendritic Cell Subtypes for Pro-Inflammatory Cytokines
Source: Front Immunol. 2020 Oct 14;11:524826. doi: 10.3389/fimmu.2020.524826 (PMC7591743; doi:10.3389/fimmu.2020.524826)
Supplement: Supplementary file 3 [file DataSheet_3.pdf]

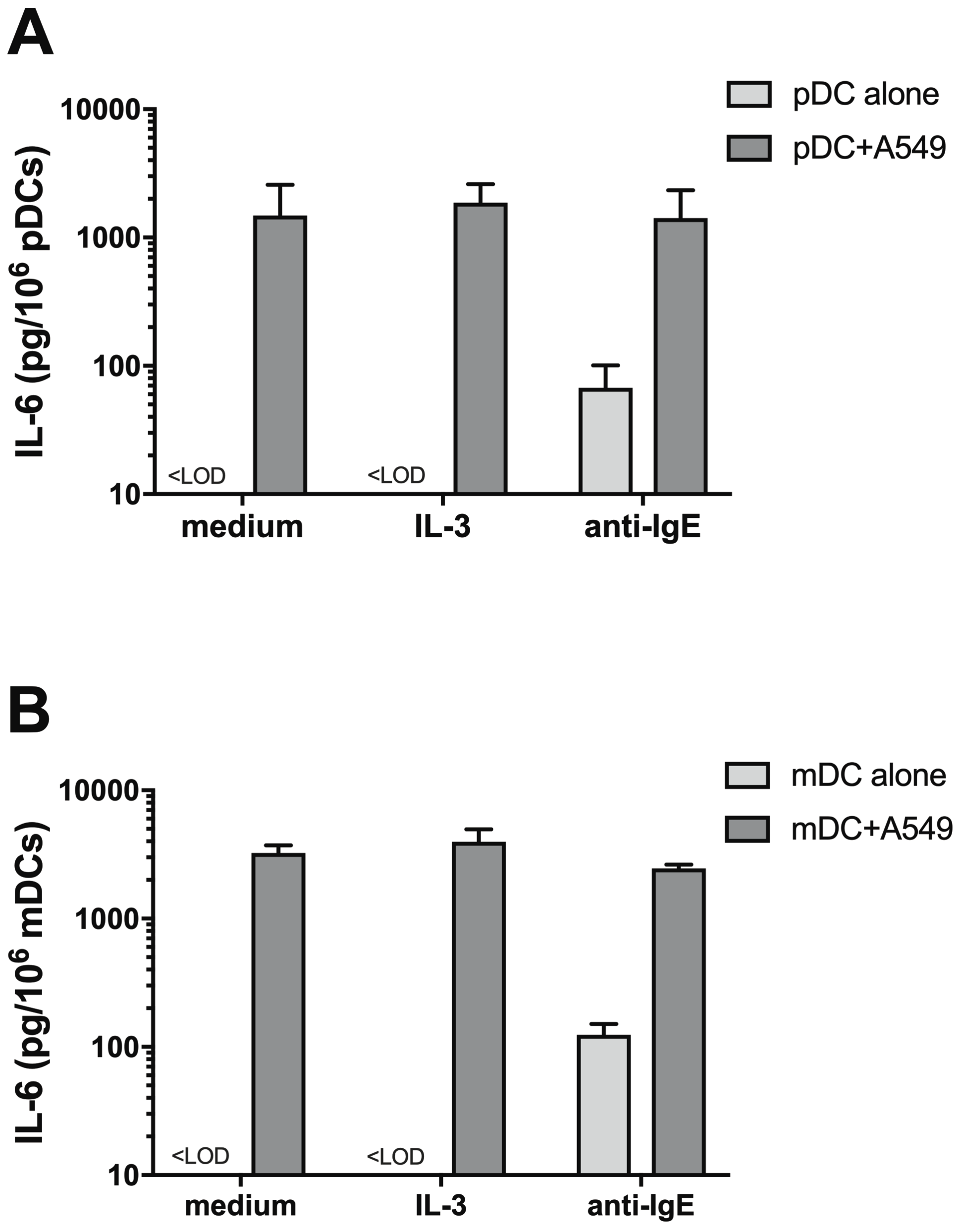

Figure S3. Responsiveness of DC subtypes (isolated by negative selection) co-cultured with A549 EC. Blood pDC and mDC were isolated using negative selection protocols and then cultured with and without A549 EC, as described in the *Materials and Methods*. Shown are the mean±sem IL-6 responses (as measured by ELISA) from co-cultures with ***A***, pDC (n=4) and ***B***, mDC (n=3) and the indicated co-stimuli, IL-3 (10 ng/ml) and anti-IgE (5 μg/ml). LOD denotes level of detection.
